# From pandemics to preparedness: harnessing AI, CRISPR, and synthetic biology to counter biosecurity threats

**DOI:** 10.3389/fpubh.2025.1711344

**Published:** 2025-11-26

**Authors:** Michael Ben Okon, Okechukwu Paul-Chima Ugwu, Chinyere Nneoma Ugwu, Fabian Chukwudi Ogenyi, Dominic Terkimbi Swase, Chinyere Nkemjika Anyanwu, Val Hyginus Udoka Eze, Jovita Nnenna Ugwu, Saheed Adekunle Akinola, Regan Mujinya, Emeka Godson Anyanwu

**Affiliations:** 1 Department of Biochemistry, Faculty of Biomedical Sciences, Kampala International University, Ishaka, Uganda; 2 Department of Publication and Extension, Kampala International University, Ishaka, Uganda; 3 Department of Microbiology and Immunology, Kampala International University, Ishaka, Uganda; 4 Department of Electrical, Telecommunication and Computer Engineering, Kampala International University, Ishaka, Uganda; 5 Department of Microbiology and Parasitology, College of Medicine and Health Sciences, School of Medicine and Pharmacy, University of Rwanda, Butare, Rwanda; 6 Department of Physiology, Equator University of Science and Technology, Masaka, Uganda; 7 Department of Anatomy, Faculty of Biomedical Sciences, Kampala International University, Kampala, Uganda

**Keywords:** biosecurity threats, global health preparedness, antimicrobial resistance, digital health, surveillance systems, global collaboration

## Abstract

Biosecurity threats, which include natural outbreaks, laboratory accidents, and intentional bioterrorism, are a major issue for global health security. The impact of poor preparedness on the health, social, and economic effects of the 1918 influenza pandemic, the 2001 anthrax attacks, and the COVID-19 crisis is devastating. Standard methods, such as quarantine and serology, as well as traditional inoculations, offered basic defences but were often reactive, slow, and unfair. The recent scientific and technological progress has altered the concept of biosecurity preparedness by providing new instruments of early detection, quick reaction, and fair health solutions. Artificial intelligence-based epidemic prediction, next-generation sequencing, CRISPR-based diagnostics, and digital epidemiology are emerging technologies that enable near-real-time surveillance. New therapeutic agents and vaccines, such as mRNA and DNA platforms, monoclonal antibodies, and nanobody therapies, have enhanced response capabilities. Containment measures based on robotics, biosensors, nanotechnology-based PPE, and portable biocontainment units have simultaneously improved frontline safety. Sensitive health information and enhanced coordination are today secured with the help of digital and cyber-biosecurity tools. Nonetheless, the innovations have ethical, legal, and equity issues, which point to the need to govern responsibly and make them accessible to all. This review brings forth the incorporation of emerging technologies with international cooperation, fair systems, and responsive policies as the keys to developing resilient and future-orientated systems that could help alleviate natural, accidental, and intentional biosecurity threats.

## 1 Introduction

Biosecurity threats pose one of the most time-sensitive issues of the contemporary world, as a broad range of biological threats can undermine the health security of the world population (1). They can be divided into broad categories, namely natural events, including pandemics due to new or re-emerging infectious diseases; accidental events, including laboratory malfunctions, mishandling of research, or unintentional release of pathogens; and deliberate events, including bioterrorism, or the intention to abuse biotechnology (2). Both categories are potentially associated with extensive morbidity, mortality, and disruption of society, whose impacts are not limited to the health systems but also extend to the economic stability, political trust, and international security (2). The increased globalisation of societies and the high rate of travelling make localised cases of biosecurity outbreaks even more dangerous, as they could turn into worldwide crises (1).

Traditionally, biosecurity threats have proven themselves many times in terms of their ability to disrupt society and overwhelm the public health systems (3). The influenza pandemic of 1918 claimed millions of lives, which still impacts the infrastructure in the field of health (4). The 2001 anthrax attacks in the United States in the 21st century demonstrated the risks of bioterrorism, as well as revealed weaknesses in preparedness (5). The recent COVID-19 pandemic has given a startling insight into how fast new pathogens can cross national borders, with disastrous health, economic, and social impacts (6). On the same note, the 2014–2016 Ebola outbreak in West Africa demonstrated major flaws in health systems, the international response, and the capacity to respond at the global level (7). Even laboratory-related accidents, despite their rarity, have underscored the risks associated with inadequate biosafety and biosecurity protocols. The following historical examples also stress the magnitude of the effect of biosecurity threats on the whole world and the necessity of active preparedness.

Global health preparedness is essential because it can enhance resilience to foreseen and unforeseen biosecurity incidents (1). Preparedness refers to the ability to avert, identify, and address biological threats by having strong surveillance, communication networks, quick diagnostic tools, and strong healthcare systems (8). Strong health systems can reduce the immediate impact of outbreaks, as well as safeguard against the long-term impact of undermining trust, stability, and development (9). Additionally, preparedness also needs to be beyond national borders since a biological threat is by definition transnational and requires a combination of international efforts (8). The inequalities experienced in crises in the past, especially those that are resource-constrained, underscore the necessity of fair access to tools and innovations that can further improve global preparedness. The purpose of this review is to determine how new technologies and innovative solutions can revolutionise global health preparedness and responses to biosecurity threats. This study evaluates the ability of technological innovation to solve long-standing preparedness challenges by analysing progress in diagnosis, surveillance, vaccine development, and digital health tools. Additionally, the review compares these innovations to traditional methods, highlighting their contrasting advantages, constraints, and implications for global equity. However, eventually, the analysis is expected to prove that the incorporation of the latest technologies, backed by effective governance and global collaboration, could be instrumental in improving resilience to natural, accidental, and intentional biological incidents and securing a safer and more secure global health future.

## 2 Global landscape of biosecurity threats

Biosecurity threats refer to a broad spectrum of biological hazards that are challenging to human, animal, and environmental health. These threats are complex and occur due to the natural processes, human activity, or intentional incorrect use of biological agents. Knowing how they are classified, trend amplification, real-life realisations, and the international structures that are geared towards curbing them are vital in the development of robust global health systems (see Table 1). We can categorise biosecurity risks as four broad categories: infectious diseases, synthetic biology threats, bioterrorism, and antimicrobial resistance (AMR).

**Table 1:** Summary of key biosecurity threats.

Case study	Type of threat	Location/Time	Impact	Key lessons
COVID-19 Pandemic	Natural infectious disease	Global, 2019–present	Millions of deaths, widespread morbidity, economic disruption, and overwhelmed health systems	Rapid spread of novel pathogens, inequities in access to vaccines and care, need for global coordination, and rapid diagnostics
Ebola Outbreak	Natural infectious disease	West Africa, 2014–2016	>11,000 deaths, long-term social/economic disruption	Fragile health systems amplify outbreaks, the importance of early detection, international support, and community engagement
Anthrax Attacks	Deliberate bioterrorism	United States, 2001	5 deaths, 17 infections, major public fear, and disruption	Even small-scale deliberate events can cause disproportionate social and psychological impact, highlighting the need for preparedness and rapid response
Antimicrobial Resistance (AMR)	Slow-moving biosecurity threat	Global, ongoing	Millions affected, rising treatment failures, projected 10 million deaths annually by 2050	Need for stewardship programs, global surveillance, and equitable access to novel antimicrobials
SARS Outbreak	Natural infectious disease	Asia, 2002–2003	~800 deaths, rapid global spread	Importance of rapid outbreak detection, information sharing, and coordinated international response
Laboratory Accidents (e.g., Smallpox and Tularemia) (118, 119)	Accidental biosecurity threat	Various	Localized infections, potential pathogen release	Critical need for biosafety and biosecurity measures, training, and regulation in laboratories

### 2.1 Infectious diseases

The most common and most impactful biosecurity risks are naturally occurring outbreaks. An infectious disease may be defined as a disease caused by a pathogen or its toxin, which occurs when an infected individual, an infected animal, or an inanimate object contaminated with the pathogen transmits the disease to a vulnerable host (10). The global burden of disease caused by infectious diseases has been of unimaginable weight to the global health systems and economies, and its negative effect is disproportionately experienced by vulnerable populations (10). The determinants of this infectious disease are the exposure of a potential host to an infectious agent; the result of the exposure is determined by the dynamic relationship between determinants of infectiveness, pathogenicity, virulence of the agent, and determinants of susceptibility to infection and disease of the intrinsic host. Extrinsic determinants that increase the host’s vulnerability to exposure include environmental factors, which consist of both physical and social behavioural elements (11). There are pathogens, including influenza, coronaviruses, and haemorrhagic fever viruses, that can infect a large number of people quickly and overload the health fields (11). New threats are a significant percentage of emerging infectious diseases, which are often of zoonotic origin (12). Infectious diseases have to be properly diagnosed to treat patients and conduct prevention and control surveillance (11). Sensitivity and specificity are two crucial attributes that any diagnostic test used should have (11). Sensitivity is the test’s ability to detect those infected (positive for disease). A very sensitive test will be more likely to identify those with the disease (and maybe those without the disease); a very sensitive test will have few false negatives (10). Specificity: This aspect is the capacity of the test to correctly recognise people who are not infected by a given agent (a healthy negative); high specificity means that there is a low number of false positives. Commonly, screening tests are highly sensitive (to detect any potential cases), and confirmatory tests are more specific (to rule out false-positive screening tests).

In general, the laboratory diagnosis of infectious diseases is founded on the tests that either directly detect an infectious agent or are indicative of infection that has occurred and show evidence of agent-specific immunity in the host (10). Diagnosis of an infecting agent may be done by direct testing of host samples (e.g., blood, tissue, urine) or environmental samples or by testing after agent culture and isolation of host samples. The principal types of analyses applied in identifying pathogens can be divided into phenotypic, which reveal the properties of an intact agent; nucleic acid-based, which identify the characteristics and composition of an agent’s nucleic acid (DNA or RNA); and immunologic, which identify the presence of an immune response to an agent (11).

### 2.2 Synthetic biology risks

Genetic engineering and synthetic biology made remarkable breakthroughs in medicine and biotechnology and brought about biosecurity issues (13). Synthetic viral genomes, pathogen virulence, and the design of resistant organisms bring dangers of unintended and intentional abuse. Examples of various emerging risks identified in synthetic biology include artificial intelligence misuse and biological dataset targeting, hacking of insecure internet-of-medical-things, genetic blackmail, and bio-discrimination (14). These risks highlight the dual-use nature of synthetic biology, where powerful tools for innovation can also be weaponized or exploited. Dual-use research of concern (DURC) is a constructive example of the ethical and security dilemmas of the advanced life sciences. There has been some progress with the synthetic biology tools used to design and optimise biological systems. Examples of transcriptional tools include synthetic promoters or RNA-based transcriptional regulation and are commonly used in the very specific control of gene expression (15). These modifications can be done through the extension of flanking sequences upstream and downstream of core promoters and by increasing the number of copies of promoters so that the desired transcription efficiency is obtained (16). It has been demonstrated by numerous studies that the translation initiation site of mRNA, including a ribosome-binding site (RBS) and the 5′ structural region (5′), makes significant contributions to the establishment of the translation efficiency of a certain mRNA (15). A research study by Hewett et al. (17) suggests 44 risks in synthetic biology, and they can be divided into four risk categories concerning human health and environmental pollution. Allergies, antibiotic resistance, carcinogens, and pathogenicity or toxicity are the problems in human-health-related risks, and environmental risks include changes in the environment or depletion, horizontal gene transfer, and pathogenicity or toxicity (17). In addition, CRISPR/Cas9, the new technology of genome editing, has had immense impacts on the sphere of synthetic biology. This technology not only enhances the accuracy and efficiency of editing of pathogens, animals, plants, and human genomes, but also produces traceless modification of genomes within a short time. Hence, the technology can increase the pathogenicity, virulence, or transfer of toxins or bacteria, or interfere with the key genes in humans, animals, and plants (18). Moreover, some research states that CRISPR/Cas9 has off-target effects, which may lead to unspecified health effects (19, 20). Additionally, facilitated and low-cost operations enhance the risks of deliberate abuse. The CRISPR/Cas9 genome-editing technology was mentioned in a report by the U. S. Intelligence Agency submitted to the U. S. Senate in 2016 as a possible weapon of mass destruction (21).

### 2.3 Bioterrorism

The intended release of pathogens or toxins, whether political, ideological, or military, is one of the gravest biosecurity threats (22). Bioterrorism incidents are not as frequent as natural outbreaks, but they may cause disproportionate fear and social destabilisation. A systematic review conducted by Elgabry et al. (23), underscores bioterrorism as a significant future threat enabled by synthetic biology, particularly through the misuse of genome editing tools like CRISPR and the increasing accessibility of biological data and technologies. It highlights how synthetic biology could facilitate the creation of more virulent pathogens or covert biological attacks, such as engineered viruses that mimic natural deaths, making detection and attribution difficult. Thirty-three terrorist attacks involving biological agents were recorded between 1970 through 2019 (24). Despite numerous possible pathogens that can be utilised in a bioterrorist attack, the most alarming agents to national security and the health of the population reported include anthrax, smallpox, plague, tularaemia, botulism, and the viral haemorrhagic fevers, in decreasing order of probability, as presented in Table 2 (22). Although the 1997 outbreak of *Shigella dysenteriae* type 2 among laboratory workers also listed in Table 2, was not officially classified as a national security threat, it underscored the potential risks posed by insider threats and intentional misuse of biological agents within controlled environments (25). The Centres for Disease Control and Prevention (CDC) classify these pathogens as Category A (26). Thus, one of the possible preventive or severity-minimising measures is vaccine prophylaxis, which is only relevant when applied before the onset of the symptom, preferably within 4 days of exposure (26).

**Table 2:** Showing proportional distribution of the main biological agents based on documented modern-era cases.

Biological agent	Type	Proportion of total bioterrorism incidents (%)	Reported incidents (*n*)	References
*Bacillus anthracis* (Anthrax)	Bacterium	38%	10–15 major events (Amerithrax, Japan incidents, etc.)	(120)
Ricin toxin	Plant-derived toxin	23%	5–10 major events (U. S. letter cases, 2013 Dutschke).	(121)
Botulinum toxin	Bacterial toxin	18%	5–7 failed attempts (Aum Shinrikyo, etc).	(122)
*Shigella dysenteriae* type 2	Bacterial pathogen	4%	1–2 events (1997 *Shigella dysenteriae* type 2 USA).	(25)
*Salmonella enterica* (Typhimurium)	Bacterium	3%	1 major event (1984) Rajneeshee food contamination (USA).	(123)
Other agents (hoaxes, e.g., Steve Kurtz case (2004) or unconfirmed, e.g., plague, smallpox threats)	Mixed	14%	Multiple cases (mostly threats, no agent used)	(124)

Reported Incidents (n): The number of incidents that were officially documented or widely reported in public records, databases, or peer-reviewed sources. Proportion of Total Bioterrorism Incidents (%): The relative share of bioterrorism incidents involving each agent, as a percentage of the total bioterrorism incidents reported from 1990 to 2019.

### 2.4 Antimicrobial resistance (AMR)

Antimicrobial resistance (AMR) is a serious worldwide biosecurity issue that has created significant problems in the health of people, economic prosperity, and efficacy of contemporary medicine (27). AMR poses a threat to the efforts made to fight infectious diseases by characterising it as the capacity of microorganisms, including bacteria, viruses, fungi, and parasites, to develop mechanisms that diminish the effectiveness of antimicrobial treatments (28). AMR is among the top 10 global public health threats identified by the World Health Organisation (WHO) (27). In 2019, AMR was directly responsible for 1.27 million deaths and contributed to an additional 3.68 million deaths globally (29). The AMR rates have also been a cause of alarm in 2020, as WHO published data on the surveillance of 78 countries (30). The seriousness of the situation is supported by the fact that the World Bank assessment estimates that the failure to curb AMR may result in the world witnessing a 1.1 per cent to 3.8 per cent decrease in global domestic product (GDP) by the year 2050 (31).

**Figure 1:**
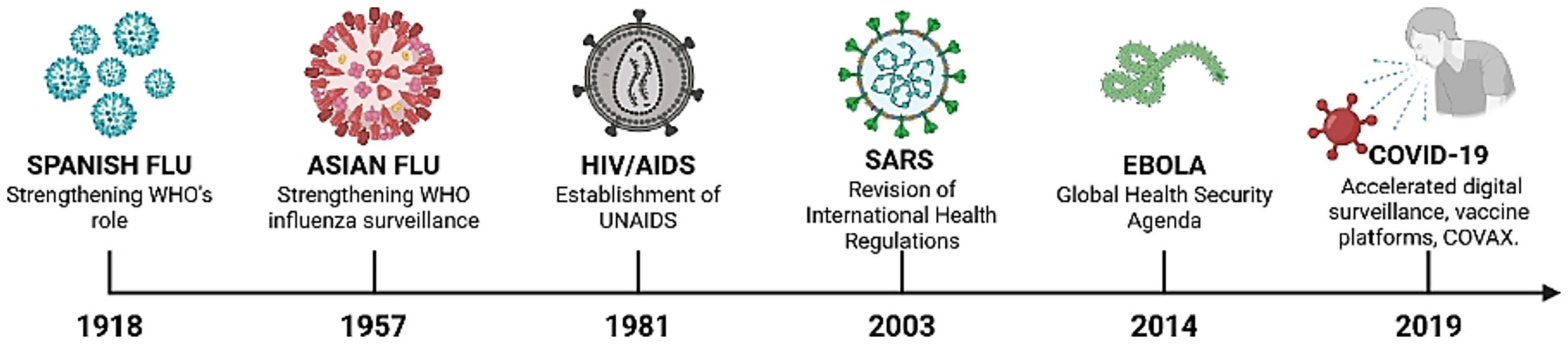
Timeline of major global outbreaks and innovation milestones in biosecurity preparedness (1918–2019).

A historical approach to major outbreaks worldwide can help offer important insights into how biosecurity policies and preparedness approaches evolved (Figure 1). The timeline outlines major events from previous pandemics, such as SARS, flu outbreaks, and Ebola, up to the recent COVID-19 crisis, and shows how each outbreak led to innovations in surveillance, diagnostics, containment, and governance (32–37). Analysing these milestones reveals how the lessons learnt from past crises have shaped current frameworks and led to the development of new technologies for anticipating and addressing future biosecurity threats.

## 3 Conventional and traditional approaches

Before the emergence of high-tech biotechnological and digital solutions, the preparedness for biosecurity threats in the world depended on traditional approaches to these problems (38, 39). These methodologies, despite their fundamental nature, were often reactive and restricted. Reactive outbreak management characterised much of the 20th century (40). The mobilisation of resources by the public health systems was usually done only after it was confirmed that an outbreak had occurred, in most cases through the clinical identification of clusters of unusual illnesses. The use of quarantine, isolation, and restrictions on borders was common, including during historic cholera outbreaks and the 1918 influenza pandemic (41). Although these measures occasionally impeded the spread of the disease, they hardly prevented any large-scale spread because of the delay in detection and lack of predictive capability. Laboratory confirmation was based on conventional investigations, including culture-based techniques and serological tests (42). Culture methods enabled the cultivation and determination of the pathogens, which revealed useful information on antimicrobial susceptibility (42). The immune responses were discovered with the help of serology, which is often applied retrospectively to verify the exposure (43). Such approaches, however, demanded the services of skilled staff, special infrastructure, and quite a considerable period of time, which could be days, weeks, or months before one could see a result. These delays were harmful in the case of fast-spreading outbreaks. Standard vaccines and antibiotics were pivotal in prevention and treatment, with notable achievements of traditional vaccines, developed from inactivated or attenuated pathogens, particularly evident in the global eradication of smallpox and polio, marking significant milestones in public health. The mid-20th century saw the introduction of antibiotics, which revolutionised the treatment of bacterial infections and significantly reduced mortality (28). However, antibiotics had long development cycles, were expensive to produce, and were only effective against known pathogens. Though traditional methods laid the groundwork for infectious disease control, their inefficiency became more evident when new and complicated biosecurity challenges came into the limelight. Lagging in detection and response was a recurrent weakness. Culture and serological diagnostics used long processing times, and in most cases, outbreaks were confirmed late. Interventions were already established in the form of transmission chains, as observed during the initial stages of the HIV/AIDS crisis and SARS outbreaks (44). The most recent and advanced method now is the integration of culturomics, which is a high-end microbiological approach that integrates high-throughput culturing with genomic sequencing to address the shortcomings of the conventional biosecurity approach, including the use of culture-based diagnostics (45, 46). In contrast to traditional methods, where the diversity of microbes, including those that are difficult to grow in culture or impossible to grow in culture, is usually greatly underestimated, culturomics maximizes the diversity of microbes by employing diverse culture conditions and incorporates molecular profiling by means of sequencing, which facilitates the detection of hitherto unidentified pathogens. It is an all-encompassing strategy that benefits biosecurity through better surveillance, outbreak surveillance and antimicrobial resistance (AMR) surveillance. Despite its capability to detect pathogens more easily and precisely, culturomics has to overcome such drawbacks as high price, infrastructure demands and complexity of data management, though its implementation into biosecurity systems becomes a strong tool in response and detection of new biological threats (45).

Conventional means could not always cope with the demands of new health threats. The development of vaccines with traditional platforms might require years, which will make them useless in the case of rapidly spreading pandemics (47). Discovery of antibiotics slowed dramatically at the end of the 20th century, and abuse and overuse contributed to antimicrobial resistance (AMR) (28). The limited laboratory facilities, human resources, and supply chains in resource-restricted areas further limit the usefulness of such methods. Traditionally, national organisations dominated the control of infectious diseases, with minimal data exchange and international cooperation (48). This piecemeal response compromised the world’s ability to respond promptly, as it took place during the initial transmission of Ebola in West Africa (2014–2016) and highlighted the poor coordination of influenza surveillance before the establishment of the WHO Global Influenza Surveillance and Response System (GISRS).

## 4 Emerging technologies in biosecurity preparedness

**Figure 2:**
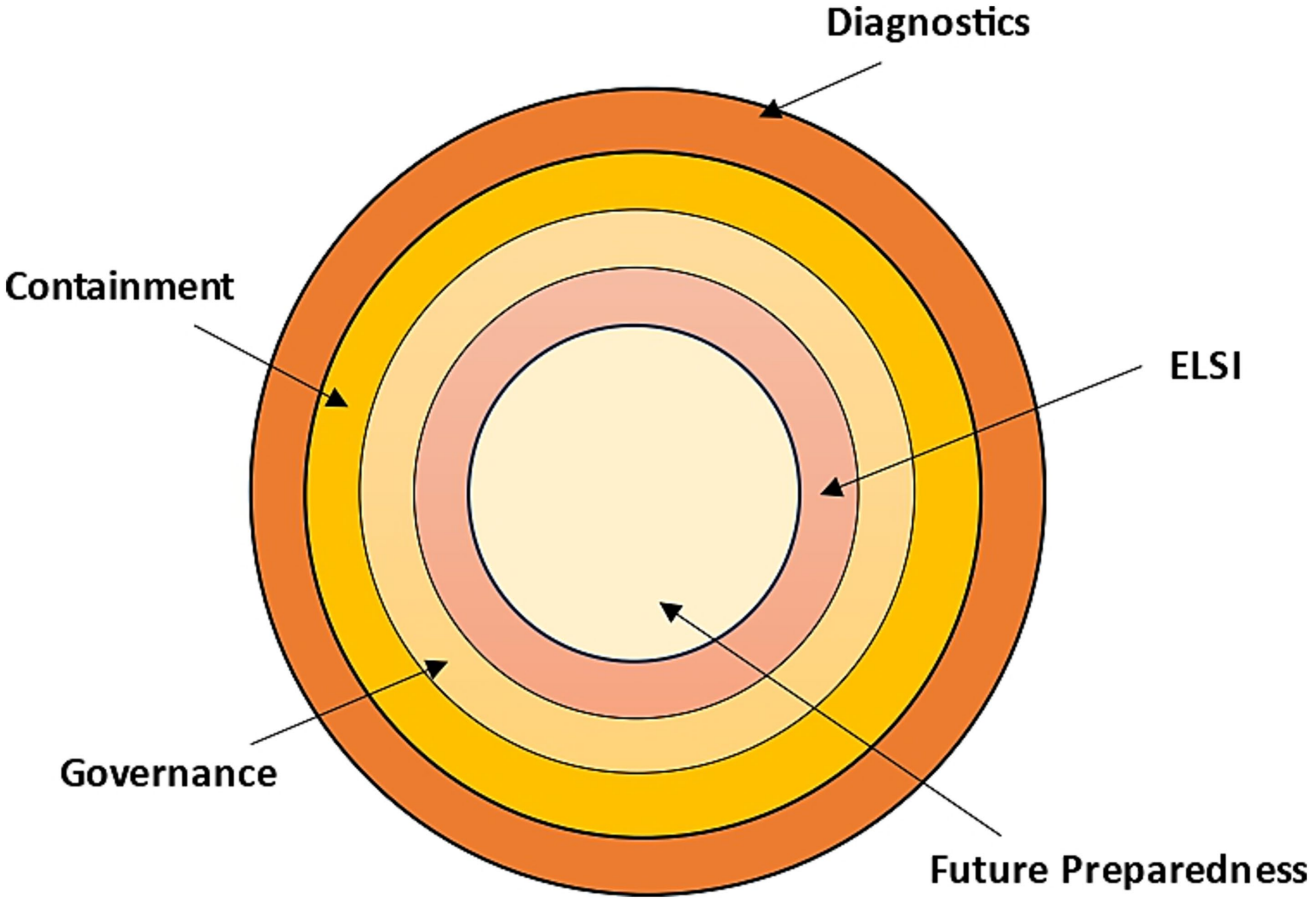
Showing the layers of biosecurity preparedness.

Recent developments in science and technology are transforming the way the global community prepares, detects, and responds to biosecurity threats. Epidemic forecasting based on artificial intelligence, next-generation sequencing, CRISPR-based rapid assays, and digital epidemiology platforms are some of the tools used in diagnostics and surveillance to detect outbreaks faster and more accurately (49, 50). In the therapeutic and vaccine sector, new technologies, such as mRNA or DNA vaccines, monoclonal antibodies, AI-assisted drug discovery, and designs made possible by synthetic biology, are hastening the creation of countermeasures to known and emerging pathogens (19, 51, 52). Containment and response technologies, i.e., biosensors, wearable health monitoring, robotics in outbreak areas, and advanced protective materials, are equally important for increasing the safety of the frontline and minimising human risk (53, 54). Simultaneously, digital and cyber-biosecurity tools, such as blockchain to exchange health data safely (55), BAKE to identify criminally-exploitable vulnerabilities in electronic devices, especially medical products (56), the Internet-of-Medical-Things (IoMT), which is transforming healthcare through the introduction of internet-connected medical-grade devices that are integrated to wider-scale health networks to improve patients’ health (57), cloud-networked emergency strategies (58), and more robust cybersecurity systems to protect sensitive health data, are increasingly important to protect sensitive health information and avert the misuse of technology. Furthermore, adequate biosecurity preparedness requires the consolidation and sequential fortification of science, systems, ethics, and foresight, as shown in Figure 2. Together, these innovations have never been used to offer such opportunities to enhance global health preparedness, but they come with the challenge of equity, safety, and governance (59), that should be handled with caution.

### 4.1 Diagnostics and surveillance

Proper diagnostics and surveillance are central to the national and world biosecurity preparedness, which allows detecting new pathogens early, describing their features, and implementing evidence-based measures to reduce the spread. Recent technological developments, especially the use of artificial intelligence (AI) to forecast threats, genomics and next-generation sequencing (NGS), CRISPR-based rapid diagnostics, and digital epidemiology based on extensive behavioural and mobility data, have revolutionised the ability to detect threats almost in real time and react to them (60). Integrating these modalities into interoperable surveillance systems is essential for transforming the traditional practice of public health into a proactive risk mitigation approach.

Now, AI-based modelling and forecasting integrate machine learning with mechanistic epidemiological models to enhance accuracy, interpretability, and strength in changing epidemic conditions (61). Modern methods integrate data-centric ML (time-series forecasting, ensemble models, deep learning) with mechanistic epidemiological models (SEIR-type compartmental frameworks) to enhance interpretability and strength in nonstationary epidemic contexts (60). These hybrid architectures minimise the error in forecasting by investigating prior mechanistic knowledge but learn complex, data-driven trends (seasonality, change of behaviour, variants) (61). Findings from a recent series of reviews and applied studies indicate that there is an enhanced lead time for resource allocation and the prediction of hotspots when AI-augmented systems are operationalised within public health pipelines (62). Significant weaknesses include data quality and representativeness, early epidemic curve overfitting, explainability of models, and cross-population predictive performance equity, which have to be overcome to achieve operational credibility (61). Some of the considerations that are applicable in practice involve transparent model validation (hindcast/real-time evaluation), ensemble forecasting to reflect epistemic uncertainty, the use of nontraditional covariates (mobility, wastewater signals, and weather), and continuous calibration as the dynamics of pathogens and human behaviour change. The regulatory and governance frameworks of AI in the field of public health must require the documentation of the model, the evaluation of bias, and the system to update the model quickly based on the new phenotypes of pathogens (63).

The further development of genomics and next-generation sequencing (NGS) also restructured the concept of pathogen surveillance by providing the capability to characterise pathogens on a genome scale, identify pathogens, perform phylogeny, and identify mutations that are linked to transmission advantage or resistance to multiple drugs (44). Large-scale use throughout the COVID-19 pandemic revealed the strength of genomic epidemiology in the detection of variants, the reconstruction of the process of their transmission, and the provision of information regarding vaccines and treatment. Mobile sequencing platforms and simplified bioinformatics pipelines have increased genomic surveillance in the field and low-resource environments, but disparities in sequencing capacity across the world remain. Genomic data can be enhanced with epidemiological metadata (timing, location, clinical severity), which enhances the investigation of outbreaks and assists in decision-making to contain and provide clinical care (64). Among the issues are turnaround times in clinical facilities, metadata standardisation, the governance of data sharing, and long-term funding for regular genomic surveillance (44). Genomic surveillance operational best practices are focused on representative sampling methods, faster sequencing-to-report pipelines (to produce actionable information within days), and interoperable data standards to facilitate interjurisdictional aggregation. To eliminate the observed capacity gaps in the lower-resource regions, it is essential to invest in workforce training and the local sequencing infrastructure (64).

CRISPR-based diagnostics (CRISPRdx) is a technology that uses programmable Cas enzymes (e.g., Cas12, Cas13) to detect nucleic acids in a sensitive and specific manner with fast readouts that can be used in point-of-care (POC) settings (50). Examples of platform exemplars include SHERLOCK and DETECTR, which are viable in viral detection during recent outbreaks and can be adapted to lateral-flow and fluorescence readouts to be used in the field (65). The benefits of CRISPRdx are high analytical sensitivity (when used with isothermal pre-amplification), short time-to-result, adaptability to retarget guides to novel pathogens or variants, and possible inexpensive production (66). Current development is aimed at enhancing single-nucleotide specificity (to differentiate point mutations), multiplexing, simple sample preparation, and non-laboratory readouts (smartphone-based imaging, handheld readers). Strong clinical-validation research and regulatory measures are hastening the bench-to-business POC test translations (50). Field applications Limitations for field applications are that a pre-amplification step is required with very low copy targets (which can complicate matters), a cold chain or reagent stability factor, and quality control and standardised performance measurements across manufacturers. The development of amplification-free CRISPR assays, lyophilised reaction chemistries, and integrated sample-to-answer cartridges can overcome these barriers (67).

Non-traditional, big-scale sources of data, such as social media activity, web search trends, call detail records, aggregated mobility metrics, and participatory symptom reporting, are also exploited by digital epidemiology to complement the traditional surveillance systems (68). These data can give earlier indications of behavioural shifts, new clusters, or even local increases in symptomatic reporting, which are the antecedents of clinical case reporting. In the COVID-19 context, several digital indicators were given early warning about the spread and monitored: the mood of the population, misinformation, and compliance with nonpharmaceutical measures (69). Digital streams, in conjunction with AI, have the potential to improve situational awareness and streamline the prioritisation of laboratory testing and targeted interventions (70). Significant ethical, legal, and technical issues include privacy, sampling bias (access to the digital divide), signal-to-noise ratio, and the reflectiveness of social media groups. Strong privacy-preserving analytics (aggregation, differential privacy), signal validation against clinical ground truth, and explicit equity evaluations are requirements for responsible adoption. In combination with both genomic and clinical information, digital epidemiology is a component of a multi-layered surveillance architecture that can identify and respond to outbreaks at an early stage (71).

The combination of AI forecasting, high-resolution genomics, rapid diagnostics using CRISPR, and digital epidemiology can act as a strong and multifaceted surveillance system with the ability to build lead time, enhance situational granularity, and target interventions (50). The implementation of this synergy would necessitate interoperable data architecture, metadata standardisation, ongoing funding for laboratory and computational capabilities (including in resource-poor environments), human resource training, and data sharing and accountability policies for AI. Pilot programmes where genomic sequencing hubs are co-located with digital signal surveillance and AI-driven early-warning systems can provide convenient avenues to assess changes in outcome measures of the response to the outbreak (time-to-detection, size of the outbreak, efficacy of the interventions). These connections will be important in enhancing the biosecurity preparedness of both naturally occurring pathogens and deliberate biological weapons (44).

Besides technological advances in biosecurity preparedness, government biosurveillance systems are also very important in identifying, tracking, and acting on biosecurity threats. The water surveillance and microbial surveillance are some of the programs needed to detect infectious diseases early and reduce their spread. An example is that the United States has several programs, such as the National Biosurveillance Integration Center (NBIC), that combine federal information streams to monitor the extent of transmission of pathogens and environmental hazards (72). Wastewater surveillance is a surveillance method used in this program to identify the existence of infectious agents in communities, which is crucial in the monitoring of pandemics, as in the example of the COVID-19 pandemic. Also, the Centers for Disease Control and Prevention (CDC) conduct microbial surveillance in different sectors to track antimicrobial resistance (AMR) and the development of new pathogens (73). The UK has a well-developed system of monitoring microbial threats through wastewater and air surveillance by the National Health Service (NHS) and UK Health Security Agency (UKHSA) (74). Digital epidemiology is also used in the UK, which combines both traditional and digital surveillance to evaluate the risk to biosecurity quickly (75). Such program integration contributes to the prompt detection of any possible outbreak, as was the case in the UK response to *E. coli* outbreaks and avian influenza.

### 4.2 Therapeutics and vaccines

The therapeutic and vaccine technologies have also improved biosecurity preparedness around the world tremendously. Recent advancements in mRNA and DNA vaccine technologies, monoclonal antibodies, nanobody therapies, AI-aided drug repurposing, and synthetic biology for vaccine design have been critical.

The development of mRNA vaccines has revolutionised the vaccinology sphere with their fast developmental paths and solid immune responses. The effectiveness of mRNA vaccines against COVID-19 has prompted interest in using such technology against other infectious diseases. Recent research has also shown the effectiveness of mRNA vaccines in preclinical disease models of diseases such as the Zika virus and cytomegalovirus and that the technology also has potential beyond respiratory pathogens (76). The development of lipid nanoparticle (LNP) delivery systems has enhanced the stability and immunogenicity of mRNA vaccines, thereby making mRNA vaccines usable across various populations and contexts (77). DNA vaccines, although not the leading counterparts of their mRNA colleagues, have some benefits regarding stability and portability, especially in low-resource environments. Recent trends aim to enhance the immunogenicity of DNA vaccines through the use of improved adjuvants and improved delivery systems, such as electroporation. DNA vaccines against such diseases as West Nile virus and malaria have demonstrated positive outcomes in clinical trials, meaning that they can be used as a complement to mRNA vaccines (78).

The monoclonal antibodies (mAbs) have also emerged as a new pillar in the treatment of a wide variety of diseases, including cancers and infectious diseases. The latest innovations resulted in the creation of bispecific antibodies, which are able to bind two antigens at the same time and increase the therapeutic effect. Clinical uses have also been extended to treat diseases like HIV, where mAbs are used to neutralise the viruses and inhibit their transmission (79). Nanobodies are the tiniest functional fragments of antibodies found in camelid species; they have been recognised as a new therapeutic modality. Their distinctive characteristics, such as small size, stability, and easy production, qualify them as viable candidates for attacking intracellular pathogens and cancer cells. Nanobodies have recently been proven effective at neutralising SARS-CoV-2 and suppressing tumour growth in preclinical models. They are in clinical trials to determine their human safety and effectiveness (80).

Artificial intelligence (AI) has also advanced drug discovery by enabling the analysis of large volumes of data to identify potential therapeutic agents. AI-driven drug repurposing has accelerated the identification of available compounds for the treatment of novel diseases. As an example, machine learning models have been utilised to forecast the effectiveness of FDA-approved drugs against SARS-CoV-2, which resulted in the quick discovery of promising drug candidates to be used in clinical trials (81). In addition, AI has been used to design new therapeutics through the structure–activity relationship between compounds and the optimization of drug properties. Recent developments in generative models have allowed the design of small molecules with targeted biological activities that simplify the drug development process. Such AI-based solutions can lower the cost and time to develop new therapies and introduce them to patients faster (49).

Similar to other approaches, synthetic biology has offered novel paradigms in vaccine development because it has made it possible to design novel antigens and delivery systems. These have found recent uses such as engineering live attenuated vaccines with better safety profiles and developing self-amplifying RNA vaccines capable of generating stronger immune responses with lower doses (52). Protein engineering and glycan engineering have also advanced and made it easy to design vaccine antigens, which are more stable and immunogenic. The innovations have resulted in the production of vaccines with a more extended coverage of the different strains of pathogens to overcome the challenges of antigenic variation (82). These innovations provide additional opportunities for preventing and treating infectious diseases, as well as increasing biosecurity preparedness on a global level.

### 4.3 Containment and response

Response and containment strategies are essential aspects of biosecurity preparedness, as they make it possible to deal with infectious threats as quickly as possible and reduce transmission. Innovations in biosensors, wearable health monitoring, robotics, nanotechnology-enabled PPE, and portable biocontainment systems have revolutionised the ability to detect, isolate, and control infectious agents at the clinical and field levels (83). The combination of these technologies promotes interventions in time, safeguards the frontline responders, and improves the efficiency of operations in case of outbreaks.

The wearable biosensors have become the key players of real-time health monitoring, providing continuous and non-invasive measurements of physiological parameters. Recent progress has resulted in the creation of miniaturised and flexible sensors that can be used to detect the presence of biomarkers of infectious diseases, including volatile organic compounds, in exhaled breath (53). By identifying the disease onset early, these machines enable us to implement timely interventions and enhance the response to the outbreak. Furthermore, biosensor data have been enhanced by incorporating machine learning algorithms, which have helped to better predict and improve the accuracy of these systems to personalise health monitoring and early warning systems (84). The clinical and community environments can transform the current methods of surveillance and health response by implementing wearable biosensors. Robotic systems have also been incorporated in the control of infectious disease outbreaks, especially under circumstances where human beings are restricted by the risks of contamination. Automated robots with artificial intelligence (AI) have performed tasks such as wastewater monitoring, pathogen detection, and environmental disinfection (85). Medical supply transportation, patient care, and facility decontamination also utilise robotics. Such autonomous systems lower the human contact with infectious agents, improve the efficiency of operations, and promote the preservation of the necessary services in case of an outbreak (54).

Designing superior PPE with the use of nanotechnology has greatly enhanced the safety of healthcare workers and responders in the case of an outbreak of an infectious disease. Antimicrobial properties include nanomaterials (like silver nanoparticles and graphene oxide) that are effective in supplementing the protective garments, masks, and gloves (86). New technologies, such as the introduction of sensors into PPE to track environmental conditions and find violations in real time, offer dynamic protection and minimise the risk of infection (87). These innovations are also used in the evolution of intelligent PPE that can react to the different levels of threat.

Portable biocontainment units (PBCUs) have been created to transport patients with high-consequence infectious diseases (HCID) long distances safely (88). These are self-contained systems with negative pressure, which employ HEPA filtration and are compatible with different means of transport, such as ground and air transportation. Simultaneously, portable decontamination systems based on the technologies of multi-spectral ultraviolet (UV) light, plasma, and others have been proposed to neutralise pathogens in contaminated regions within a short period of time (89). These systems are very effective in the deactivation of a wide range of microorganisms, such as bacteria, viruses, and fungi, and thus, improve the containment and the response capacity to outbreaks.

### 4.4 Digital and cyber-biosecurity tools

Increased healthcare and biotechnology digitisation has brought about opportunities, as well as vulnerabilities in biosecurity. Digital and cyber-biosecurity solutions, including blockchain for secure data sharing, robust cybersecurity measures to support bioinformatics and synthetic biology, and cloud-based health emergency platforms, enable the real-time and safe management of sensitive health information. Such tools increase coordination, mitigate the abuse of biological data, and improve the resilience of cyber threats in the public health and outbreak management systems.

The adoption of blockchain technology has continued to ensure the safety of health data exchange with regard to data integrity, privacy, and interoperability. Blockchain can guarantee a secure and unaltered registry of health data because it offers a decentralised system that cannot be modified by a third party (55). The most recent applications involve hybrid blockchain systems that integrate both public and private chains to trade off transparency and confidentiality and enable a safe exchange of data across healthcare systems and increase trust in stakeholders (90).

Synthetic biology has been met with artificial intelligence (AI), with new biosecurity issues, such as the possible misuse of bioinformatics tools and the development of novel pathogens. Cybersecurity is necessary to protect against unauthorised access to genetic data, the malicious design of harmful biological agents, and the responsible use of biotechnology (91). The measures to address risks include the adoption of effective cybersecurity measures, the creation of safe bioinformatics solutions, and the creation of management systems to control the use of synthetic biology in an ethical manner (92).

Cloud computing has also revolutionised medical emergency management by offering scalable and flexible platforms for data storage, analysis, and sharing. Cloud-based needs allow the real-time tracking of health indicators, the synchronisation of responses, and the sharing of information in case of outbreaks (58). The latest developments are the implementation of Internet of Things (IoT) devices with cloud infrastructure to enable continuous health monitoring and the application of edge computing to localise data processing to minimise latency and improve the responsiveness of health emergency platforms (93).

It is also notable that the development of global health preparedness represents a gradual process of transitioning between the use of traditional tools and the reactive approaches to the innovations aimed at speed, flexibility, and integration. Although each of the two methods helps to learn valuable lessons, the comparative analysis of the two approaches indicates that there are essential insights to be made regarding the future of biosecurity preparedness as presented in Table 3.

**Table 3:** Comparative analysis of conventional vs. emerging approaches in biosecurity preparedness.

Dimension	Conventional approaches	Emerging approaches	Synergies	Barriers/Challenges
Diagnostics & Surveillance	Culture-based tests and serology are often slow (days to weeks)	CRISPR-based, point-of-care tests; AI-driven surveillance; real-time genomic sequencing	Conventional tools remain the gold standard for confirmation, while emerging tools enable rapid detection and early alerts	High cost of sequencing; AI data privacy concerns; limited infrastructure in LMICs
Prevention & Mitigation	Standard vaccines (inactivated/attenuated); antibiotics	Next-gen vaccines (mRNA, DNA, nanoparticles); synthetic biology; phage therapy & novel antimicrobials	Conventional vaccines provide long-term immunity; new platforms offer speed and adaptability	Unequal vaccine access; antimicrobial resistance; dual-use risks of synthetic biology
Response & Containment	Quarantine, isolation, manual supply delivery, traditional PPE	Digital health & telemedicine, drones, robotics, wearable biosensors, Internet-of-Medical-Things (IoMT), culturomics and BAKE	Emerging tools enhance reach and efficiency while conventional measures provide fundamental safeguards	Cost of robotics/drones; mistrust in digital tools; weak digital infrastructure in some regions
Systemic Capacity	Reactive outbreak management; fragmented global coordination	Proactive surveillance, predictive modelling, and integrated health networks	Combining strong systems with innovative tools ensures agility and resilience	Sustainability of funding, lack of skilled workforce, and technology gaps between regions
Equity & Access	Limited global sharing; reliance on national responses	Global data-sharing (GISAID), COVAX, Pandemic Fund, CEPI-led collaborations	Systemic reforms + innovation foster inclusive preparedness	Risk of widening inequities; dependency on donor financing; weak governance
Ethical & Security Concerns	Few ethical debates historically (focused on safety)	AI surveillance, genomic data use, synthetic biology → major dual-use risks	Governance frameworks can balance innovation with oversight	Dual-use bioterrorism risks; lack of international consensus on bioethics

## 5 Global collaborative frameworks

Biosecurity preparedness is based on global collaboration, which allows countries and organisations to coordinate their efforts, exchange important information, and share resources during health emergencies. The WHO, UN agencies, and regional health networks are institutions that have major roles in streamlining the efforts and implementing international health regulations, as well as establishing public-private partnerships that enhance the speed of biotechnology innovations and enhance global governance.

Global health security has its coordinating centre at the World Health Organisation (WHO), which leads the global health response to disasters and establishes global health standards (94). The United Nations (UN) facilitates the work of WHO by assisting in the efforts of the former through different special agencies that focus on the more comprehensive determinants of health, such as socioeconomic factors and environmental conditions (95). Regional health networks, including the WHO Regional Health Alliance (RHA), are a solution that enables countries that are neighbours to jointly tackle any health-related issues that cross countries, to share resources and knowledge and respond to health crises (96). Instead, the International Health Regulations (2005) (IHR) are legally binding rules that establish the rights and responsibilities of countries in managing public health events and emergencies that may have international consequences (97). Although the IHR is expected to increase global health security, problems remain with its practical implementation, such as poor reporting, insufficient surveillance, and resource mobilisation in low-income nations (97). These loopholes support the necessity of more powerful compliance systems and the assistance the countries need to fulfil their duties in accordance with the IHR.

Public-privacy partnerships (PPP) in the context of biosecurity refer to collaborations between government agencies (public sector) and private companies or organizations to enhance public health preparedness in promoting biotechnology innovation, especially in the design of diagnostics, therapeutics, and vaccines. The collaborative efforts include public institutions, private industry, and academia in research and development in areas of unmet needs in public health (98). Such collaborations complement the capabilities of both sectors to hasten the process of turning scientific breakthroughs into practical health solutions to increase global readiness for new infectious disease outbreaks. Example of this PPP is the Biosecurity Leadership Council (BLC) which is now renamed as the Engineering Biology Responsible Innovation Advisory Panel (RIAP) (Engineering Biology Responsible Innovation Advisory Panel - GOV.UK) plays a pivotal role in fostering public-private partnerships (PPPs) to enhance the UK’s biosecurity preparedness, GAVI, which help to increase access to immunization in low-income countries by facilitating the procurement and distribution of vaccines (99), and Coalition for Epidemic Preparedness Innovations CEPI’s whose aim is to accelerate the development of vaccines for emerging infectious diseases (100).

Global biosecurity heavily depends on the effective data sharing that will allow the timely identification of health threats and informed decisions. Privacy issues, national security, and various regulatory standards frequently constrain information exchange (101). International agreements and governance are being developed to address these challenges, ensuring that data-sharing practices are harmonised to facilitate responsible and ethical data sharing while protecting individual rights and national interests.

## 6 Case studies

COVID-19 demonstrated both the potential and limitations of global preparedness for health emergencies. Another strength was the record-breaking vaccine development, especially the use of mRNA platforms in the year of the outbreak (102). International organisations like COVAX tried to make vaccine distribution equitable, but still, there were inequities, and low-income countries got their vaccines much later (103). Among the weaknesses were delays in the global reporting, lack of coordination among the countries, and lack of necessary protective gear, which indicated that more resilient supply chains and greater adherence to the International Health Regulations (IHR) were required (104).

CRISPR-based diagnostics, like SHERLOCK and DETECTR, have become quick, portable, and very sensitive to detect SARS-CoV-2 and its variants (105). These technologies enabled decentralised testing, particularly in low-resource environments where traditional PCR testing facilities were scarce. Their low cost, scalability, and the ability to respond to a variety of pathogens proved their usefulness in pandemic preparedness, but there are still problems related to regulatory approval, manufacturing capacity, and field validation (50).

Predicting Ebola epidemics through the use of epidemiological, mobility, and ecological information has seen more extensive use of artificial intelligence (AI) and machine learning (ML) models (106). Table 4 summarises these predictive systems, enabling early detection of high-risk areas for resource allocation and preparedness ahead. Nevertheless, challenges include issues such as the lack of data, the need for correct mobility tracking, and the necessity of integrating predictive tools into national public health systems (107). The mRNA vaccines (Pfizer-BioNTech and Moderna) have been successful, which is a milestone in biotechnology. Several decades of previous research, synthetic biology, and international public-private collaborations allowed them to grow fast (51). The innovation established new standards of speed in clinical trials and international regulatory collaboration. Significantly, mRNA manufacturing can be scaled, which will provide future vaccines against emerging pathogens, such as influenza, RSV, and possible pandemic threats (47).

**Table 4:** Case studies in global biosecurity preparedness (2020–2025).

Case study	Key strengths	Key weaknesses/challenges	Implications for biosecurity	References
COVID-19 Global Response	Rapid development of mRNA vaccines; global initiatives (COVAX).	Inequitable vaccine access, fragile supply chains, and delays in international reporting.	Need for resilient supply chains, stronger IHR compliance, and equitable distribution mechanisms.	(102–104)
CRISPR-Based Diagnostics	Portable, rapid, and sensitive; enabled decentralized testing in low-resource settings.	Regulatory approval delays, limited scalability, and manufacturing capacity.	Integration into surveillance networks and scaling production for future pandemics.	(50, 105)
AI in Ebola Prediction	Machine learning predicted outbreak hotspots using epidemiological and mobility data.	Data scarcity, reliance on accurate real-time data, and limited integration into health systems.	Investment in real-time surveillance and AI-driven early warning tools.	(106, 107)
Rapid mRNA Vaccine Deployment	Demonstrated record-breaking vaccine development and scalability, supported by PPPs and synthetic biology.	Manufacturing concentration in high-income countries; inequitable access in LMICs.	Expand decentralized vaccine manufacturing hubs and sustain PPP-driven innovation.	(47, 51)

## 7 Ethical, legal, and social considerations (ELSI)

New developments in synthetic biology and gain-of-function studies pose two-sided dilemmas of dual-use research, in which a study aimed at benefiting society can be used maliciously to harm individuals (108). Ethical systems focus on openness, safety of biology, and control systems, yet the management of risky studies is not balanced worldwide (108). The problem is to arrive at a compromise between the freedom of science and the prevention of bioterrorism threats. The COVID-19 pandemic revealed the existence of severe disparities in terms of access to vaccines. Even though the high-income nations gained access to early supplies, in the majority of the low-income areas, both the purchase and introduction of vaccines were delayed (109). There were efforts like the COVAX that aimed to reduce inequities, but structural barriers remained. To achieve equity, it is necessary to have decentralised vaccine manufacturing centres in Africa, Asia, and Latin America (110). Biotechnology is rapidly developing, and regulatory regimes do not keep pace. Although the experience of COVID-19 and fast-tracked approvals of vaccines showed the advantages of adaptive regulation, there are still concerns about the long-term safety and proper oversight (111). Regulatory organisations need to come up with strong, adaptable policies that embrace innovation without compromising safety or ethics. The problem of misinformation and disinformation during COVID-19 had a devastating impact on the vaccination campaigns and the people’s adherence to health measures (112). Open communication about risks, community involvement, and collaboration with trusted local leaders is necessary to address this. We should regard the establishment of trust among people as the foundation of biosecurity preparedness (113).

## 8 Future directions and policy recommendations

The key to the further development of global biosecurity and the preparedness for new threats is strategic foresight and consistent policy action. The future of the issues is based on strengthening international governance structures, investing in resilient and fair health systems, responsible biotechnology development, and education and civic involvement (1). Particularly the education and civic engagement are central to enhancing biosecurity and preparedness on a global scale, as well as making communities informed and engaged that can respond efficiently to the threat of a biological attack. Educational programs especially those with a focus on biosecurity and disaster preparedness increase the knowledge of people and help to encourage risk-reducing behaviors. As an example, the studies have shown that disaster preparedness education has a considerable positive impact on the resilience of the community, providing individuals with the knowledge and skills required to effectively respond to emergencies (114). Besides, civic engagement practices, including community-based disaster preparedness interventions, have been reported to increase social cohesion and collective efficacy, which are key to effective biosecurity responses (115). Moreover, the incorporation of civic education in curricula has been linked with the rise in the number of people participating in civic health programs and an enhanced responsibility to the community, which enhances the biosecurity response at the community level. All of these educational and civic engagement measures will help to create resilient societies that could effectively prevent, detect, and respond to biological threats. Effective coordination and response to any health emergencies can be achieved by reinforcing international agreements, especially the International Health Regulations, and improving compliance mechanisms. Simultaneously, to ensure early identification and efficient control of biological risks, it is critical to develop strong health systems by investing in infrastructure, developing a workforce, and building capacity. The capacity to ensure fair access to health services and technologies is key to the reduction of disparities and universal health coverage and, consequently, increases the resilience of different populations.

New biotechnologies, such as gene editing and synthetic biology, have transformative potential in the field of diagnostics, therapeutics, and vaccines, but their development should be informed by strict safety measures and ethics to reduce the chances of misuse or undesirable side effects (116). It is also significant to develop a skilled and competent workforce via education and training and to develop public participation to create trust, transparency, and active participation of people in biosecurity activities (117). Collectively, these measures can help not only to make biosecurity preparedness scientifically sound but also ethically sound and socially inclusive and, therefore, to create a more resilient and secure global health environment.

## 9 Conclusion

The international response to the threats posed by biosecurity should no longer be a reactive response but a proactive strategy. The new technologies, including the use of AI in forecasting, diagnostics using CRISPR, and mRNA, provide prompt detection and reaction, but their usefulness requires equal access, ethical control, and powerful governance. We must strengthen health systems in resource-constrained areas and globally to promote resilience and equity. The population’s trust, open communication, and community interaction further enhance preparedness. The world can create inclusive systems that will reduce natural, accidental, and intentional biological threats and ensure a safer global health future by using scientific innovation, equity, and global cooperation.
